# Evaluation of temperature rise in the pulp during various IPR techniques—an in vivo study

**DOI:** 10.1186/s40510-020-00340-6

**Published:** 2020-11-02

**Authors:** Kiran Banga, Nitin Arora, Sridhar Kannan, Ashish Kumar Singh, Abhita Malhotra

**Affiliations:** Manav Rachna Dental College, Sector 43, Suraj Kund, Badkhal Road, Faridabad, Haryana 121004 India

**Keywords:** Orthodontics, Interproximal enamel reduction, Pulp temperature, IPR kit

## Abstract

**Background:**

Non-extraction treatment protocol has gained a lot of popularity over extraction for orthodontic treatment. Interproximal enamel reduction is one such method that makes it possible to do orthodontic treatment without extractions. This procedure, which can be done by various techniques, leads to a rise in the temperature of the pulp of the teeth. Previously, studies have been done which have evaluated the temperature changes inside the pulp chamber of extracted teeth, during interproximal enamel reduction. However, no documented literature exists that has evaluated these changes in the live pulp of the teeth whilst interproximal enamel reduction (IPR) is being performed. Therefore, this study aimed to evaluate the temperature changes inside the live pulp of the teeth during various interproximal enamel reduction techniques in vivo.

**Aims:**

Evaluation of temperature rise in the pulp during various interproximal enamel reduction techniques, done in vivo.

**Material and method:**

The study was performed on patients for whom extraction of premolars had been advised for their orthodontic treatment. Fifty-one premolar teeth were randomly divided into three groups of IPR, i.e. using airotor and bur, handheld metal strip and orthodontic IPR kit (oscillating system). IPR was performed on the mesial and distal sides after access opening, temperature change was recorded during IPR and the readings were compared. The Shapiro-Wilk test was utilized for checking whether the data satisfied the requirement of normal distribution.

**Results:**

The highest temperature rise was seen in group 1 in which interproximal enamel reduction was performed using airotor and bur. The minimum temperature rise was observed in group 2 in which interproximal enamel reduction was done using the handheld metal strip, whereas the temperature rise observed in group 3, in which interproximal enamel reduction was done using IPR kit, was between the range of group 1 and group 3. The temperature change was in the following order—group 1 (2.08 °C) > group 3 (1.22 °C) > group 2 (0.52 °C).

**Conclusion:**

None of the methods used to perform interproximal enamel reduction caused a temperature increase more than 5.5 °C, beyond which pulp necrosis may occur. Therefore, all three methods used in the study for IPR were found to be safe.

## Background

With the current paradigm shift in orthodontics, interproximal enamel reduction (IPR) has gained popularity over extractions, for comprehensive orthodontic treatment. Interproximal enamel reduction technique or IER, out of many, is one method to gain space to relieve crowding in the arches. It is defined as “a clinical procedure that requires the proximal enamel surfaces to be reduced, anatomically re-contoured for the correction of any inconsistency in the tooth shape” [1]. The other commonly used terminologies for this procedure are “stripping,” “re-approximation,” “slenderization,” “coronoplastia,” “slicing,” “mesio-distal reduction,” “selective grinding” and “Hollywood trim.”

Dr. Charles H. Tweed propounded the universal objectives of comprehensive orthodontic treatment as “esthetically pleasing, healthy, functional and stable occlusion, which should esthetically match the harmony of the soft tissue profile” [2]. To obtain these standards is sometimes difficult, especially in patients where excess tooth material is found to be interfering with the optimal alignment of their teeth, as excess tooth material has been identified as an aetiology of malocclusion [3]. This, in return, becomes the fons et origo of crowding of the teeth, as a result of the tooth size versus arch length discrepancy, which is one of the most common types of malocclusion encountered by an orthodontist [3].

IPR is primarily indicated for the reshaping of the proximal contact [4, 5], for solving the Bolton discrepancy [1], for treating mild to moderate crowding, for reducing interdental gingival papillary retraction and for stabilizing the dental arches [6]. Enamel stripping can also be done in patients with an indication of Frankel I or II appliance, in cases where the deciduous molar needs to be retained when there is a congenital absence of the succedaneous premolar [7, 8], and lastly, to reduce or prevent the formation of black triangles amongst the teeth.

Clinicians, over the years, have propagated various methods to carry out this procedure, out of which, the ones that can be deemed most common are manual abrasive strips, diamond-coated segmented discs, rotating diamond burs and mechanical oscillating abrasive strips [6, 9].

Even though this procedure is used routinely in an orthodontic office, there are certain drawbacks of IER, which one should take into consideration. All rotary cutting instruments produce heat and mechanical vibration that can harm the pulp of the tooth [10–12]. The heat, if transferred to the pulp, can lead to histopathological changes and can also cause necrosis of the pulp [13–15]. Therefore, Zachrisson [16, 17] and Sheridan [18] put great emphasis on the use of a coolant substance whilst performing this procedure.

Visibility is another indispensable factor whilst performing the IER procedure. Proper access and visibility are imperative in order to avoid periodontal tissue injuries and also to prevent scarring of the proximal enamel [19]. Conventional polishing methods have failed to remove enamel surface injuries [20]. Radlanski et al. [21, 22] noted the formation of furrows in the posterior enamel surfaces because of improper stripping, resulting in an increase of plaque accumulation.

It is advised to use wires, elastics, separators, coil spring, etc. to obtain a smooth proximal surface, natural morphology of the tooth and to prevent ledges whilst performing IER procedures [23]. The use of water coolant, and suction at times, may make the interdental enamel reduction procedure a tedious task, obstructing the vision of the operator and also causing discomfort to the patient. This may lead to this procedure becoming a four-hand task.

Zach and Cohen, in 1965, reported in their study conducted on rhesus monkeys that temperature rise beyond 5.5 °C leads to pulp necrosis [24]. Baysal et al. [19] evaluated the temperature changes inside the pulp chamber during various IER procedure, without using water coolant in vitro. They noted a major rise in temperature using a high-speed airotor with a tungsten carbide bur and stressed upon the need for a continuous application of coolant.

Pereira et al. [25] also evaluated the changes in temperature, without using cooling substances for this procedure. They used extracted incisors for their study. Perforated stripping discs and handheld stripper were used, and they observed a significant difference in pulp temperature during these stripping techniques too. However, the rise in temperature was found to be below the critical threshold in all groups.

Apart from these studies, there is exiguous scientific literature in which thermal changes in the pulp during various IER procedure have been evaluated. Previously, all the studies evaluating these changes have been done in vitro, on extracted teeth and devoid of live pulp. No scientific evidence exists that narrates the changes in temperature inside the live pulp tissue, during interproximal enamel reduction procedures. Therefore, the purpose of this study was to evaluate in vivo the temperature changes in the pulp during various enamel reduction techniques.

## Method

As per our knowledge, no previous in vivo study has been done to assess the changes in temperature during IPR in the pulp; therefore, a pilot study was done in the Department of Orthodontics & Dentofacial Orthopaedics, Manav Rachna Dental College, Haryana, India, on 15 patients, in which the mean temperature rise after access opening was calculated. After access opening was done, the temperature change was noted at 1-min intervals till the temperature became constant and maintained or till the pulp cooled off after getting heating due to access opening. The mean time taken for the temperature to become constant in the pilot study was 5 min post-access opening, which was taken as the time at which the baseline temperature of every premolar was recorded in the main study. After the baseline temperature was reached, IPR was performed on the premolars to note the temperature rise in the pulp, in the main study. The mean baseline temperature was 36.7 °C. This also served as the control group.

The source of collecting data for the study was the maxillary and mandibular premolars of patients for whom extraction of premolars had been advised for orthodontic treatment. The study was performed in the Department of Orthodontics & Dentofacial Orthopaedics, Manav Rachna Dental College, Haryana, India.

A minimum total sample size of 51 (17 per group) was found to be sufficient for an alpha of 0.05, power of 80% and an effect size of 0.45 (assessed for the increase in pulp temperature from the pilot study).

The inclusion criterion is premolars without caries.

The exclusion criteria are (i) premolars with fractured crowns and (ii) premolars with pulp pathologies.

Premolars were randomly divided into three groups:

- Group 1: IPR using a airotor and bur
- Group 2: IPR using metal hand strip
- Group 3: IPR using IPR kit

**Fig. 1:**
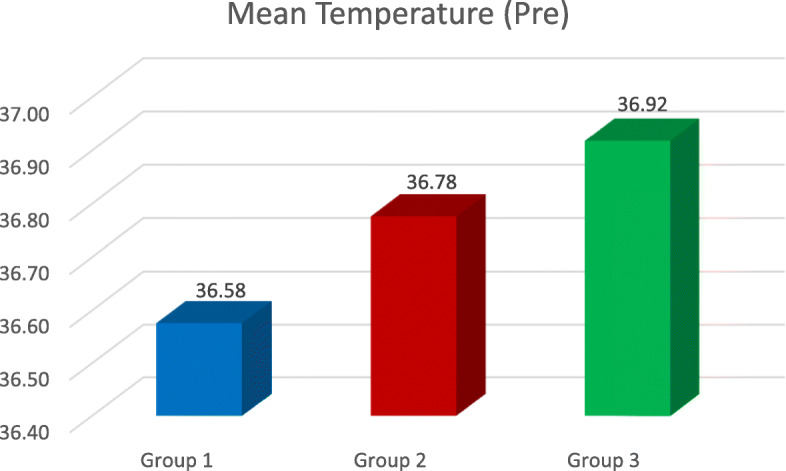
The mean temperature before IPR

The materials used were as follows:

- ▪ Digital thermometer with K-type thermocouple probe (Generic TCOUP). K-type thermocouple probe was used in this study due to its longer life span and larger temperature range (− 454 °F to 2300 °F/− 270 °C to 1260 °C).
- ▪ Handheld interproximal enamel reduction strip (Horico 4 mm, single-sided medium grit).
- ▪ Orthodontic interproximal enamel reduction kit which is one of the latest oscillating systems for IPR. It consists of a contra-angle handpiece onto which saw-type diamond oscillating IPR strips (saw type) can be attached, which move in an oscillating or a “to and from” motion.
- ▪ Airotor (NSK).
- ▪ Carbide bur (No. 859 size 010, Diatech).
- ▪ Local aneasthetic agent, 2 ml syringe.

1. After taking the informed consent from each participant, local anaesthesia was administered to them.
2. Once the local anaesthesia became effective, access opening was done on the premolar.
3. Baseline temperature was recorded after 5 min of access opening time (mean time taken for the pulp temperature to return to normal and stabilize after access opening, calculated from the pilot study) (Fig. 1).
4. After recording the baseline temperature, interproximal enamel reduction was performed on both mesial and distal sides of the tooth during which the changes in pulp temperature were recorded in degree Celsius (°C).
5. The tooth was then extracted.
6. All post-extraction guidelines were explained to the patient, and necessary medication prescription was given.

The temperature of all 51 teeth was recorded using the same methodology, and the temperature change was then compared and evaluated. The average of temperature readings on the mesial and distal sides was calculated by adding the mesial and distal temperature readings and then dividing it by 2 (Fig. 4). The resultant reading was then subtracted from the baseline temperature in order to calculate the temperature rise. Temperature changes were noted during IPR on both the mesial and distal sides as the thickness of the enamel on the distal side of the tooth is slightly more than the mesial side [3], because of which heat transfer to the pulp could vary on both the sides.

## Result

**Fig. 2:**
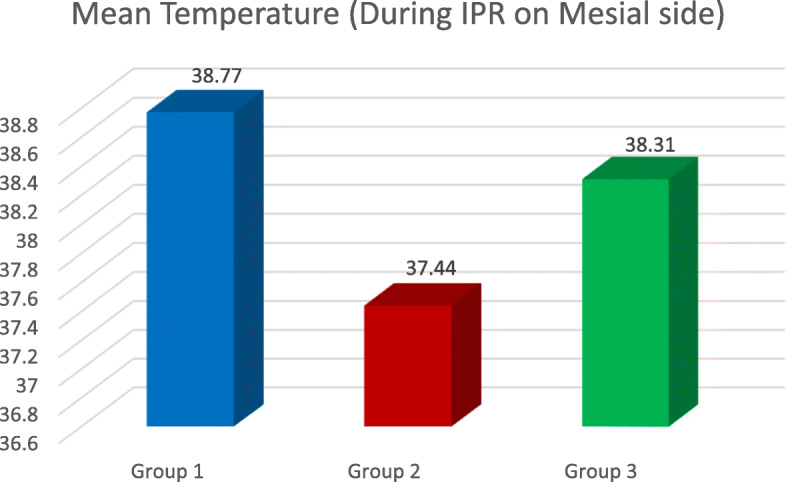
The mean temperature during IPR on the mesial side

**Fig. 3:**
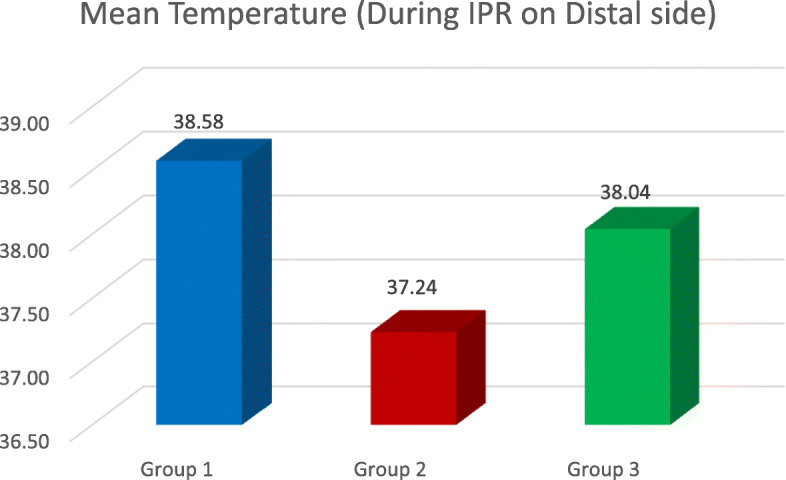
The mean temperature during IPR on the distal side

**Fig. 4:**
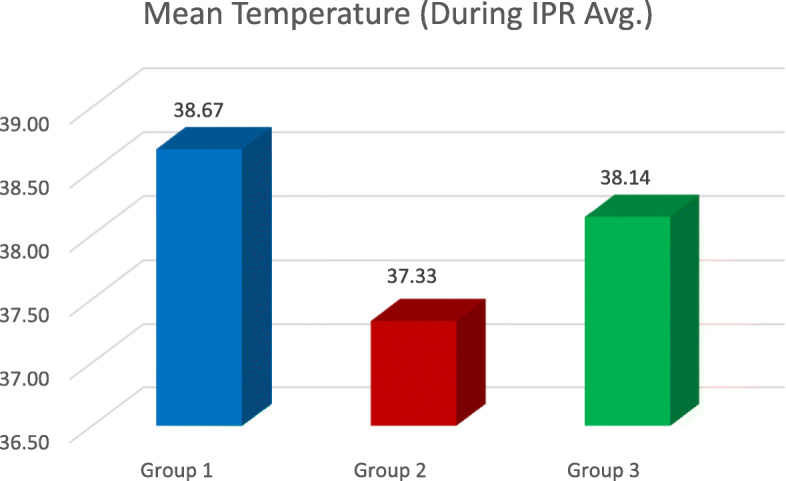
The mean temperature during IPR – the mean temperature during IPR – the average of mesial and distal readings (mesial Temp. + distal Temp. ÷ 2)

**Fig. 5:**
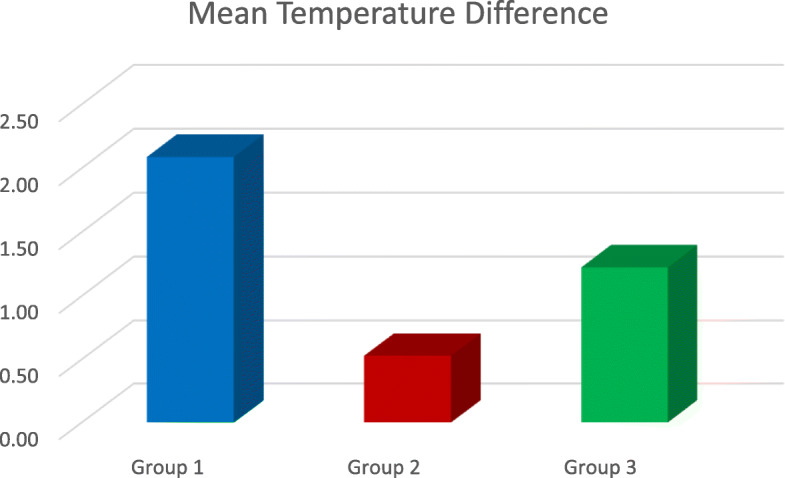
The mean temperature difference between the three groups, considering 0 °C rise in the control group

The results gave a clear picture about the change in temperature inside a live tooth pulp, during IPR. The highest temperature rise was seen whilst using airotor and bur when a comparison between all the three groups as well as with the control group was done (2.08 °C) (Table 1, Figs. 2, 3, 4, and 5). This is because the rotary cutting instrument, bur mounted on an airotor in this case, runs at a speed of 3–5 lakhs RPM, generating a lot of heat.

**Table 1:** Temperature readings in group 1 (airotor and bur)

Group	Tooth no.	Temp. before	During IPR, mesial	During IPR, distal	IPR Temp. Avg.	Temp. Diff.
1	14	37	39.9	39.4	39.6	2.6
1	44	37.4	40	40	40	2.6
1	24	37.2	39.8	39.5	39.6	2.4
1	34	37.1	40.2	40	40.1	3
1	14	35.9	38.5	38.1	38.3	2.4
1	24	35.7	38.3	38	38.1	2.4
1	34	35.4	38.1	38	38	2.6
1	44	36.4	38.7	38.5	38.6	2.1
1	34	36.8	38.8	38.2	38.5	1.7
1	24	37.2	39.7	39.4	39.5	2.3
1	44	36.4	38.5	38.4	38.4	2
1	14	35.9	37.4	37.1	37.2	1.3
1	24	36.7	38.5	38.5	38.5	1.8
1	34	37.1	39.3	39.1	39.2	2.1
1	34	35.8	37.4	37.2	37.3	1.5
1	24	36.9	38.3	38.1	38.2	1.3
1	25	36.9	38.3	38.3	38.3	1.4

The lowest temperature rise was seen whilst using metal hand strip, i.e. (0.52 °C) (Table 2, Figs. 2, 3, and 5) as this is a manual procedure in which enamel reduction is significantly slow (Livas et al.). Even though temperature rise was recorded whilst using metal hand strip, it was insignificant (0.52 °C) (Table 3, Figs. 2, 3, and 5).

**Table 2:** Temperature readings for group 2 (handheld metal strip)

Group	Tooth no.	Temp. before	During IPR, mesial	During IPR, distal	IPR Temp. Avg.	Temp Diff.
2	14	37	37.8	37.5	37.6	0.6
2	44	36.7	37.4	37.1	37.2	0.4
2	14	36.8	37.3	37	37.1	0.3
2	24	36.5	37.4	37.4	37.4	0.9
2	44	37.2	38	37.7	37.8	0.6
2	24	37.1	37.8	37.5	37.6	0.5
2	34	36.6	37.2	37	37.1	0.5
2	14	36.3	36.9	36.6	36.9	0.3
2	34	36.6	37.6	37.3	37.4	0.8
2	45	37.4	38	38	38	0.6
2	35	36.9	37.5	37.2	37.3	0.4
2	24	36.4	36.8	36.6	36.7	0.3
2	44	36.7	37.5	37.2	37.3	0.6
2	14	37.2	37.9	37.5	37.7	0.5
2	34	37	37.9	37.8	37.8	0.8
2	14	37.1	37.7	37.5	37.6	0.5
2	34	35.7	36.2	36.1	36.1	0.4

**Table 3:** Temperature readings of group 3 (IPR kit)

Group	Tooth no.	Temp. before	During IPR, mesial	During IPR, distal	IPR Temp. Avg.	Temp. Diff.
3	14	37.1	38.3	38.1	38.2	1.1
3	14	36.7	38.2	37.6	37.9	1.2
3	44	37.2	38.8	38.8	38.8	1.6
3	24	36.9	38	37.8	37.9	1
3	34	36.9	38.1	37.8	37.9	1
3	14	36.8	37.9	37.6	37.7	0.9
3	44	36.5	37.9	37.7	37.8	1.3
3	14	36.8	38	37.8	37.9	1.1
3	44	36.8	38.3	38.1	38.2	1.4
3	14	36.8	38.2	38	38.1	1.3
3	44	37.1	NA	38.5	38.5	1.4
3	14	37	38.6	38.4	38.5	1.5
3	44	36.9	38.4	38.3	38.3	1.4
3	24	37	38.2	38	38.1	1.1
3	34	37	38.3	38.1	38.2	1.2
3	14	37.1	38.4	38.1	38.2	1.1
3	14	37	38.3	38	38.2	1.2

Temperature rise using IPR kit was between the range of temperature rise seen in the other two groups, i.e. 1.22 °C (Tables 3 and 4, Figs. 2, 3, and 5). Though orthodontic IPR kit is a mechanically working system, its speed is less as compared to an airotor, i.e. 5000 RPM (Omer et al.).

**Table 4:** Temp. before IPR

Temperature before					
Group	*N*	Mean	Std. deviation	95% confidence interval for mean	
				Lower bound	Upper bound
1	17	36.576	.6220	36.257	36.896
2	17	36.776	.4146	36.563	36.990
3	17	36.918	.1741	36.828	37.007
*P* value		0.089, NS			

The results demonstrated that the rise in temperature during IPR on the mesial surface was in Gr 2 < Gr 3 < Gr 1 order (Table 5, Fig. 2), where Gr 1 is airotor and bur, Gr 2 is handheld metal strip and Gr 3 is IPR kit (*P* < 0.0001). During IPR on the distal surface, it was also in Gr 3 < Gr 2 < Gr 1 order, where Gr 1 is airotor and bur, Gr 2 is handheld metal strip and Gr 3 is IPR kit (*P* < 0.0001) (Tables 6 and 7, Fig. 3). Upon comparing the average temperature changes, the order seen was Gr 2 < Gr 3 < Gr, where Gr 1 is airotor and bur, Gr 2 is handheld metal strip and Gr 3 is IPR kit (*P* < 0.0001) (Tables 4 and 8, Fig. 5).

**Table 5:** Temp. during IPR on the mesial side

During IPR (mesial)					
Group	*N*	Mean	Std. deviation	95% confidence interval for mean	
				Lower bound	Upper bound
1	17	38.77	.872	38.32	39.22
2	17	37.44	.486	37.19	37.69
3	17	38.31	.464	38.09	38.33
*P* value		< 0.0001			
Post hoc pairwise comparison		Gr 2 < Gr 3 < Gr 1

**Table 6:** Temp. during IPR on the distal side

During IPR (distal)					
Group	*N*	Mean	Std. deviation	95% confidence interval for mean	
				Lower bound	Upper bound
1	17	38.576	.8657	38.131	39.022
2	17	37.235	.4782	36.989	37.481
3	17	38.041	.3242	37.875	38.208
*P* value		< 0.0001			
Post hoc pairwise comparison		Gr 3 < Gr 2 < Gr 1

**Table 7:** Avg. of mesial and distal temperature rise during IPR

During IPR Avg. Temp.					
Group	*N*	Mean	Std. deviation	95% confidence interval for mean	
				Lower bound	Upper bound
1	17	38.671	.8644	38.226	39.115
2	17	37.329	.4661	37.090	37.569
3	17	38.141	.2830	37.996	38.287
*P* value		< 0.0001			
Post hoc pairwise comparison		Gr 3 < Gr 2 < Gr 1

**Table 8:** Intergroup Temp. rise difference

Temperature difference					
Group	*N*	Mean	Std. deviation	95% confidence interval for mean	
				Lower bound	Upper bound
1	17	2.088	.5159	1.823	2.353
2	17	.529	.1795	.437	.622
3	17	1.224	.1921	1.125	1.322
*P* value		< 0.0001			
Post hoc pairwise comparison		Gr 2 < Gr 3 < Gr 1			

## Discussion

The evaluation of thermal changes causing pulpal damage during slenderization procedures has received very little scientific evaluation; thus, in the present study, the temperature changes in the pulpal chamber during different slenderization procedures were evaluated in vivo

In this study, the teeth with any possible structural variables that could manifest the thermal conductivity differences were eliminated. However, even after this, the teeth exhibited morphological variations in the enamel and dentin structure and thickness. Also, the teeth selected in this study did not belong to the same age group. The mineral content of the teeth as well as the size of the pulp chamber differ according to age [26, 27]. This explains the slightly different temperature values obtained amongst the teeth tested in the same group.

The baseline temperature of the pulp before IPR was noted for each tooth, post which IPR was performed. The mean baseline temperature readings were calculated for each group (Fig. 1). For performing IPR with handheld metal strips, the Horico 4 mm, single-sided medium grit were used. These are single-sided stainless steel strips, coated with the medium grit diamond and are 4 mm in width. In previous studies, strips of 6 mm width were used [19, 28].

The carbide bur mounted on an airotor (No. 859 size 010, Diatech) was used. The bur was sterilized by dry heat up to 340 °F/170 °C or autoclave up to 250 °F/121 °C.

The last slenderization procedure evaluated for the temperature change in this study is by using IPR kit which consisted of a contra-angle handpiece onto which oscillating strips were mounted. This oscillating system is one of the latest techniques in performing IPR. Not many studies have reported the use of this kit. However, Livas et al. [29] mentioned in a literature review that the use of segmented discs adapted over a shuttle head with oscillating movement has become quite popular. These discs have an advantage of better visual access. Also, Gazzani et al. [9] reported in their study that this system is more efficient in enamel reduction and also reduces the chair time.

A K-type thermocouple unit was used instead of a J-type to measure the temperature change. This was because of the high precision, reliability and wider temperature range of the K-type thermocouple, as demonstrated by previous studies [19, 30]. Although the thermocouple probe was held inside the pulp chamber, closely approximating the surface being reduced, it is, however, an arbitrary method, which is entirely manual.

The use of handheld metal strips caused a mean temperature rise of 0.52 °C. The minimum and maximum temperature rise observed was 0.3 °C and 0.9 °C, respectively. Baysal et al. [19] reported a mean temperature rise of 1.21 °C ± 1.48 °C with minimum 0.23 °C and maximum 6.26 °C. Pereira et al. [28] reported an average temperature change of 1.24 °C ± 0.3 °C and the greatest temperature rise of 1.7 °C. The results of the present study were found to be similar to these studies except for the maximum temperature change noted in the study by Baysal et al. where greater and higher change was noted than the critical threshold. In the present study, the maximum temperature rise was found to be well below the critical unlike the previous study.

The use of carbide bur in the present study showed a mean temperature rise of 2.08 °C where minimum temperature rise is 1.4 °C and maximum, 3.0 °C. Baysal et al. [19] evaluated the temperature rise in the pulp chamber using the carbide burs. They observed a mean temperature rise of 5.63 °C ± 1.73 °C with a minimum temperature change of 2.11 °C and maximum, 8.37 °C. Omer and Al Sanea [30] reported 3.5 °C as the maximum temperature rise in the pulp chamber using carbide bur. Both these studies were performed in vitro. The result of our study was nearly the same as these studies.

Upon using the orthodontic IPR kit, the mean temperature rise was 1.22 °C. The minimum and maximum temperature changes were 0.9 °C and 1.6 °C, respectively. The temperature change using this method was between the range of temperature change observed whilst performing IPR using handheld metal strips and airotor and bur. The temperature rise did not cross the threshold value of 5.5 °C. JT Blank (https://www.aegisdentalnetwork.com/id/2010/03/revolutionizing-interproximal-enamel-reduction) reported that IPR kit handpiece runs at a speed of 5000 RPM, which is significantly less than the speed of an airotor (3–5 lakhs RPM), causing less heat generation as compared to an airotor. Therefore, even though this is a mechanical procedure, the temperature rises less than that of the airotor group. None of the three groups reached the threshold value of 5.5 °C which Zach and Cohen [25] reported. All three procedures were found to be safe for performing interproximal enamel reduction.

### Limitations of the study

The present in vivo study has only observed temperature rise in premolars. The results may not be the same in the anterior teeth due to the difference in enamel thickness. In addition to this, the temperature readings were recorded manually which could have made the temperature recordings less accurate, as it is an arbitrary method. A software could be devised in the future, for more accurate readings. Lastly, upon access opening, the pulp chamber was exposed to the external environment, and temperature changes may vary in a closed pulp chamber

## Conclusion

As interproximal enamel reduction is an excellent alternative for extractions for orthodontic treatment, it is imperative to understand all aspects related to this procedure. Amongst many other factors, temperature rise in the teeth is one key feature that needs to be considered during IPR. Comparison of different slenderization procedures in this study showed least mean temperature rise with the handheld metal strips which was 0.5 °C—mean; followed by orthodontic IPR kit which was 1.22 °C—mean; and the highest was seen whilst using airotor and bur, i.e. 2.08 °C. All three procedures were found to be safe for interproximal enamel reduction.

## Data Availability

The data sets generated and/or analysed during the current study are not publicly available due to most of the journals from which the data has been taken are not open access journals and therefore require a subscription. But the data is available from the corresponding author on reasonable request.

## Abbreviations

IER: Interproximal enamel reduction
IPR: Interproximal enamel reduction
SPSS: Statistical Package for the Social Sciences
ANOVA: Analysis of variance
No.: Number
Avg.: Average
Temp.: Temperature
Diff.: Difference
Std.: Standard
NS: Non-significant
Gr: Group
